# Quantitative analysis of Robertsonian chromosomes inherited by descendants from multiple Rb heterozygotes of *Mus m. Domesticus*

**DOI:** 10.3389/fcell.2022.1050556

**Published:** 2022-11-25

**Authors:** Eliana Ayarza, Gabriel Cavada, Tamara Arévalo, Alam Molina, Soledad Berríos

**Affiliations:** ^1^ Departamento de Tecnología Médica, Facultad de Medicina, Universidad de Chile, Santiago, Chile; ^2^ Instituto de Salud Poblacional, Facultad de Medicina, Universidad de Chile, Santiago, Chile; ^3^ Programa Genética Humana, ICBM, Facultad de Medicina, Universidad de Chile, Santiago, Chile

**Keywords:** Robertsonian chromosomes, meiotic drive, meiosis, heterozygotes, *Mus domesticus*

## Abstract

Robertsonian translocation is the most common chromosomal rearrangement in mammals, and represents the type of chromosomal change that most effectively contributes to speciation in natural populations. Rb translocations involve double-strand DNA breaks at the centromere level in two telocentric chromosomes, followed by repair ligation of the respective long arms, creating a metacentric Rb chromosome. Many different chromosomal races have been described in *Mus musculus domesticus* that show reduced chromosome numbers due to the presence of Rb metacentric chromosomes*.* The crossroads between ancestral telocentrics and the new metacentric chromosomes should be resolved in the meiotic cells of the heterozygote individuals, which form trivalents. The preferential segregation of metacentric chromosomes to the egg during female meiosis I has been proposed to favor their fixation and eventual conversion of a telocentric karyotype to a metacentric karyotype. This biased segregation, a form of meiotic drive, explains the karyotype changes in mammalian species that have accumulated Rb fusions. We studied and compared the number of Rb chromosomes inherited by the offspring of multiple Rb heterozygous of *M. domesticus* in reciprocal crosses. We did not find that the Rb chromosomes were inherited preferentially with respect to the telocentric chromosomes; therefore, we found no evidence for the meiotic drive, nor was there a random distribution of Rb chromosomes inherited by the descendants.

## Introduction

Robertsonian (Rb) translocation is the most common chromosomal rearrangement in mammals (King 1993) and that most effectively contributes to variation or speciation in natural populations (Garagna et al., 2001a; Aniskin et al., 2006). In *Mus musculus domesticus,* many different chromosomal races have been described that show reduced chromosome numbers due to the presence of metacentric Rb chromosomes (Pialek et al., 2005). Rb translocations involve double-strand DNA breaks at the centromere level in two telocentric chromosomes, followed by fusion of the respective long arms, creating a metacentric Rb chromosome. The short arms (p) of the original telocentric chromosomes, including the proximal telomeres, part of the satellite DNA and, frequently one centromere, are lost (Nanda et al., 1995; Garagna et al., 2001a; Garagna et al., 2002). Most current models of chromosomal variation of natural populations assume that Rb chromosomes are negatively tolerated; only after a long period can the new chromosome could eventually overcome the meiotic restrictions and become established within a reproductive community (Baker and Bickham, 1986; Rieseberg 2001; Hauffe et al., 2012). However, six distinct chromosomal races of the house mice of the Madeira Island have emerged in less than 500 years. This remarkable example of chromosome evolution has been explained by reproductive chromosomal isolation and genetic drift (Britton-Davidian et al., 2000; Britton-Davidian et al., 2005). The crossroads between ancestral telocentrics and new metacentric chromosomes should be resolved in heterozygote individuals, wherein trivalents are formed during meiosis I (Wallace et al., 2002). The Mendelian distribution of metacentric/telocentric chromosomes in the heterozygote gametes will depend on the normal progression of the meiotic prophase, random arrangement of the trivalents at metaphase I, and alternate segregation between them at anaphase. The meiotic drive (also called segregation distortion) is the preferential selection of certain chromosomes or gametes that alters the gene ratio from the Mendelian expectations.

Although multiple Rb heterozygotes showed an increased loss of spermatocytes at prophase I, many of them entered meiotic divisions (Garagna et al., 2001b; Wallace et al., 2002; Manterola et al., 2009). The chromosomes of each trivalent move together into alignment at metaphase and separate from each other at anaphase I. Only gametes resulting from alternate segregation exhibit normal or balanced karyotypes. Gametes produced by adjacent segregations have unbalanced karyotypes, being nullisomic or disomic in one or more chromosomes. Selective mechanisms, such as the metaphase checkpoint, appear to be effective because spermatids resulting from alternate segregation prevail in heterozygotes (Eaker et al., 2001; Anton et al., 2004; Pylyp et al., 2013; Manieu et al., 2014; Lamotte et al., 2018). Additionally, the direction of alternate segregation during female meiosis I could potentially determine whether metacentric chromosomes are transmitted to the offspring (Yoshida and Kitano, 2012).

According to the meiotic drive hypothesis, Rb metacentrics segregated preferentially to the egg in populations that have fixed multiple different metacentrics; and preferentially segregate to the polar body in other populations that have remained telocentric (de Villena and Sapienza 2001; White et al., 2010). It is still unclear what determines the direction of the drive and how that directions can differ between populations, so that some of them retain the fusions and change karyotype, while others do not. The relative centromere strength of Rb metacentric *versus* homologous telocentrics has been proposed to determine the direction of the meiotic drive, with stronger centromeres preferentially remaining in the egg (Chmátal et al., 2014). In addition, asymmetries in the meiotic spindle orientation, tubulin and microtubule-organizing center, and associated meiotic drivers may contribute to explain the meiotic drive in oocytes (Akera et al., 2017; Wu et al., 2018; Kruger and Mueller., 2021).

In this study we attempted to determine whether the meiotic drive could be taking place during the meiosis of Robertsonian heterozygotes of *Mus musculus domesticus* who carry eight Rb chromosomes, by studying the number of Rb chromosomes (0–8), inherited by the descendants of parental males or females who are multiple heterozygous Rb.

## Materials and methods

### Animals and crosses

**FIGURE 1:**
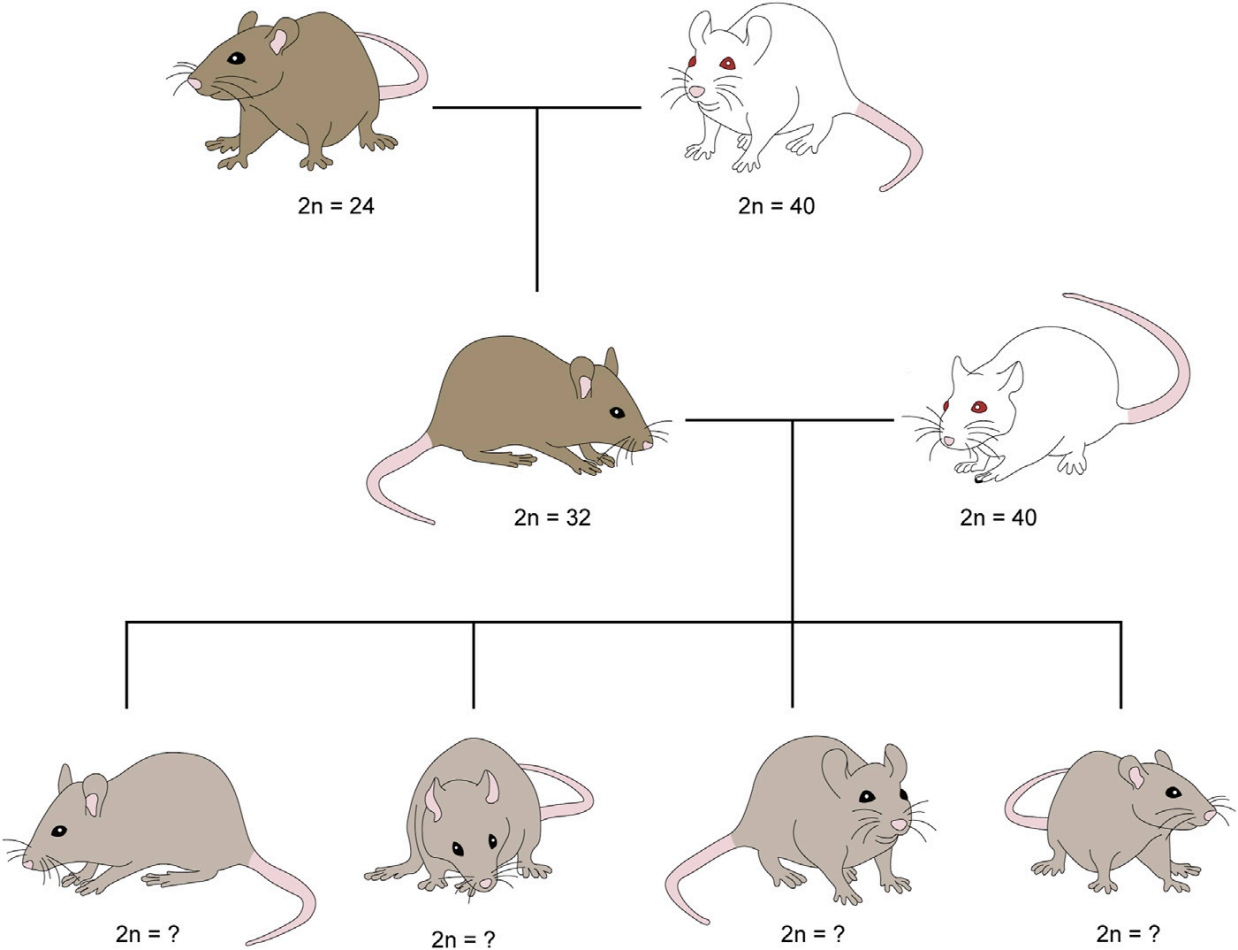
Origin of the mice under study that can inherit between 0 and 8 Rb chromosomes. Males and females of Robertsonian (Rb) heterozygotes *2n* = 32 of *Mus musculus domesticus* were generated by crossing homozygotes Milano II race *2n* = 24, with eight pairs of Rb metacentric chromosomes, and homozygote strain CD1 *2n* = 40, with all chromosomes telocentric. In turn, heterozygotes were crossed with homozygotes (*2n* = 40) to obtain offspring who could inherit between 0 and 8 Rb metacentric chromosomes.

Males and females of the Rb heterozygotes 2n = 32 group of *Mus musculus domesticus* were generated by crossing the homozygote strain CD1 2n = 40, with all their chromosomes telocentric, and the homozygote Milano II race 2n = 24, with eight pairs of Rb metacentric chromosomes. The heterozygotes were crossed with homozygotes (2n = 40), to obtain offspring who could inherit between 0 and 8 Rb metacentric chromosomes (Figure 1). The eight Rb chromosomes are: Rb 2.12, 3.4, 5.15, 6.7, 8.11, 9.14, 10.13, 16.17. Numbers correspond to the 2n = 40 standard karyotype. Nineteen crosses of heterozygous females (2n = 32) and homozygous males (2n = 40) were performed with litter, with an average of 4.4 offspring in each. Ten crosses of heterozygous males (2n = 32) and homozygous females (2n = 40) were made with litter, with an average of 5.5 offspring each.

Mice were maintained at 22°C with a light/dark cycle of 12/12 h and fed *ad libitum*. The procedures involving the use of the mice were reviewed and approved by the Ethics Review Committee of the School of Medicine, Universidad de Chile (No. CBA #0441), and by the Ethics Review Committee of the Chilean National Science Foundation FONDECYT-CONICYT. The care and handling of laboratory animals was done following all institutional and national guidelines (protocol CBA #0441, FMUCH).

### Number of Robertsonian chromosomes and size of pericentromeric region

The number of Rb chromosomes inherited by 139 descendants of female or male Rb heterozygotes was studied in metaphase plate chromosomes obtained from bone marrow cells and stained with Giemsa or DAPI. Briefly, bone marrow cells were incubated for 10 min in 0.05 M KCl at 37°C, and the pellet was washed and fixed in 3:1 v/v methanol: acetic acid. Rb chromosomes and total chromosomes were counted in 10 metaphase plates per animal. After determining the number of Rb metacentric chromosomes, we verified that the telocentric chromosomes actually completed the corresponding diploid number.

The size of the pericentromeric region was measured in the Rb chromosomes inherited by each of the five offspring of a 2n = 32 heterozygous father and a 2n = 40 mother, which were respectively: Three, five, three, two, four, and five Rb chromosomes, respectively. Chromosomes were stained with DAPI (4′, 6-diamino-2-phenylindole), which distinguishes A-T base-rich pericentromeric heterochromatin. Metaphase plates were stained for 10 min in the dark with 10 µl DAPI (Thermo Fisher Scientific^®^) in 40 µl 1x PBS buffer, washed with 1x PBS buffer, and sealed with coverslips. The samples were observed under a Nikon^®^ Optiphot fluorescence microscope and digitized using a Nikon^®^ Digital Sight DS-5M camera.

The segmented tracing tool of the Fiji software (Just ImageJ, image processing and analysis in Java) was used to measure both chromatids and thus estimate the average total length of each chromosome (TL). The length of the centromeric region (CL) was measured in pixels and normalized with respect to the chromosomal total length (CL/TLx100), to reduce errors caused by differences in chromosome compaction. For each specimen, 20 metaphase plates were studied, and the total values were recorded according to the number of Rb chromosomes present in each metaphase plate. Hence, there were 160 values for the heterozygous parent that had eight Rb chromosomes and only 40 values for the son D that had only two Rb chromosomes. The estimated pericentromeric region length of Rb metacentric chromosomes (CL/TL) for each offspring was compared with the normalized average length of the pericentromeric regions of the eight Rb chromosomes of the parental heterozygote, which was also measured in 20 metaphase plates.

### Statistical analysis

To test the null hypothesis that Rb chromosomes inherited by the offspring of multiple heterozygous parents have a random and equal distribution for the offspring of male and female heterozygous parents, a theoretical binomial distribution with probability *p* = 0.5 was generated, whose probability function is given as:

$$P\left(X=x\right)=\left(\begin{array}{c} 8\\ x \end{array}\right)0.5^{x}\;,X=0,1,2,3\ldots ,8$$

Based on this distribution, the expected frequencies of the appearance of offspring with the Rb chromosome were calculated, and the Chi-2 goodness-of-fit test was used to compare the expected frequencies with those observed. The theoretical distributions, as percentages, are shown in Table 1.

**TABLE 1:** Expected frequencies of the appearance of offspring with the 0 to 8 Rb chromosomes, calculated on the theoretical binomial distribution with probability *p* = 0.5.

Descendants according Rb chromosomes inherited	Expected descendants in binomial distribution %
0	0.39
1	3.13
2	10.94
3	21.88
4	27.34
5	21.88
6	10.94
7	3.13
8	0.39
	100.00

We used the binomial proportion test to test the null hypothesis that 50% of the offspring could have a larger pericentromeric region of Rb chromosomes than the centromeric average size of 21.51 of the eight heterozygous father Rb chromosomes. The confidence interval was 95% and 5% was used for statistical significance. The data were processed using STATA v. 16.0.

## Results

Number of Rb chromosomes present in the offspring of crosses between Rb heterozygous male or female parents and 2n = 40 homozygotes of ***Mus domesticus***.

We analyzed a total of 139 mice that were descended from crosses between heterozygous 2n = 32 with eight metacentric Rb chromosomes and homozygous 2n = 40 with all telocentric chromosomes were analyzed. Of these, 83 came from the cross of heterozygous females and homozygous males, and 56 from the crosses of heterozygous males and homozygous females (Table 2). In the chromosome set of offspring, 0 to 8 Rb metacentric chromosomes were found; however, in six offspring of heterozygous mothers, Rb chromosomes were absent, and no offspring showed seven Rb chromosomes (Table 2). The total number of Rb chromosomes present in the descendants of heterozygous females and males were 256 and 228, respectively. The average number of chromosomes per each descendants of heterozygous females was 3.08 (38.6%) and that of heterozygous males was 4.07 (50.7%), which shows significant difference (<0.01).

**TABLE 2:** Number of Rb chromosomes inherited by descendants of crosses between Rb heterozygous male or female parents and homozygotes *2n* = 40, of *Mus m domesticus* mice.

Heterozygous females			Heterozygous males		
Number of Rb chromosomes per cell	Number of descendants according Rb chromosomes	Total Rb chromosomes in offspring	Number of Rb chromosomes per cell	Number of descendants according Rb chromosomes	Total Rb chromosomes in offspring
0act	6	0	0	2	0
1	7	7	1	1	1
2	22	44	2	5	10
3	10	30	3	14	42
4	20	80	4	14	56
5	15	75	5	10	50
6	2	12	6	5	30
7	0	0	7	1	7
8	1	8	8	4	32
TOTAL	83	256	TOTAL	56	228

Roughly, the set of 484 Rb chromosomes inherited by the total descendants would be equivalent to approximately 44% of the maximum possible number of 1,112 metacentric Rb chromosomes, considering eight Rb chromosomes per 139 descendants. This suggests that although the Rb chromosomes were highly inherited, they were not preferred over telocentric chromosomes.

To assess whether the number of Rb chromosomes inherited by the offspring of multiple heterozygous parents has a random and similar distribution for offspring of male and female heterozygous parents, a theoretical binomial distribution with probability *p* = 0.5 was generated and the observed data were compared.

**FIGURE 2:**
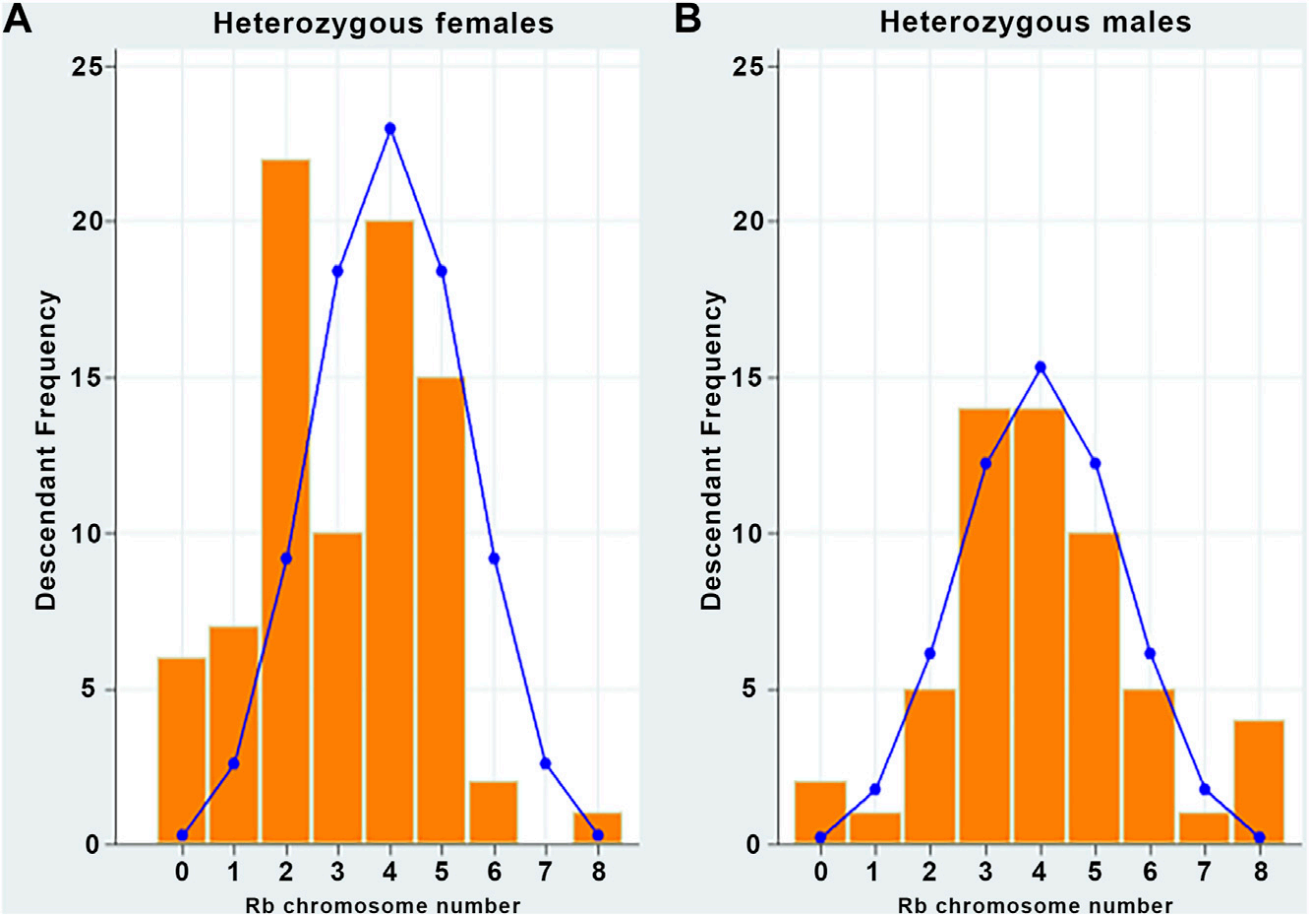
Observed frequencies do not fit the theoretical binomial distributions. **(A)** Observed (bars) descendants from heterozygous females by inherited Rb chromosome and expected (curve) frequencies according to the binomial distribution (*p*-value = 0.0000). **(B)** Observed (bars) descendants from heterozygous male by inherited Rb chromosome and expected (curve) frequencies according to the binomial distribution (*p*-value = 0.0000).

In binomial distributions, the expected frequencies of descendants from heterozygous female or male parents according to the inherited Rb chromosome are shown in Table 3, and the comparison between the observed and the expected frequencies of descendants from female and male heterozygous parents is shown in Figure 2.

**TABLE 3:** Observed number of descendants by inherited Rb chromosome and expected frequencies according to the binomial distribution. Heterozygous females (*p*-value = 0.0000); Heterozygous males (*p*-value = 0.0000).

Number of Rb chromosomes per cell	Heterozygous females		Heterozygous males	
	Number of descendants according Rb chromosomes (Expected)	Number of descendants according Rb chromosomes (Observed)	Number of descendants according Rb chromosomes (Expected)	Number of descendants according Rb chromosomes (Observed)
0	0.3	6	0.22	2
1	2.6	7	1.75	1
2	9.2	22	6.13	5
3	18.4	10	12.25	14
4	23.0	20	15.31	14
5	18.4	15	12.25	10
6	9.2	2	6.13	5
7	2.6	0	1.75	1
8	0.3	1	0.22	4
	83	83	56	56

In both cases, the null hypothesis was rejected (*p* < 0.05), which means that the data do not follow a binomial distribution pattern; therefore, they can be associated with non-random inheritance.

Pericentromeric region size in Rb chromosomes present in the offspring of crosses between heterozygous Rb males and 2n = 40 homozygous females of ***Mus domesticus***.

**FIGURE 3:**
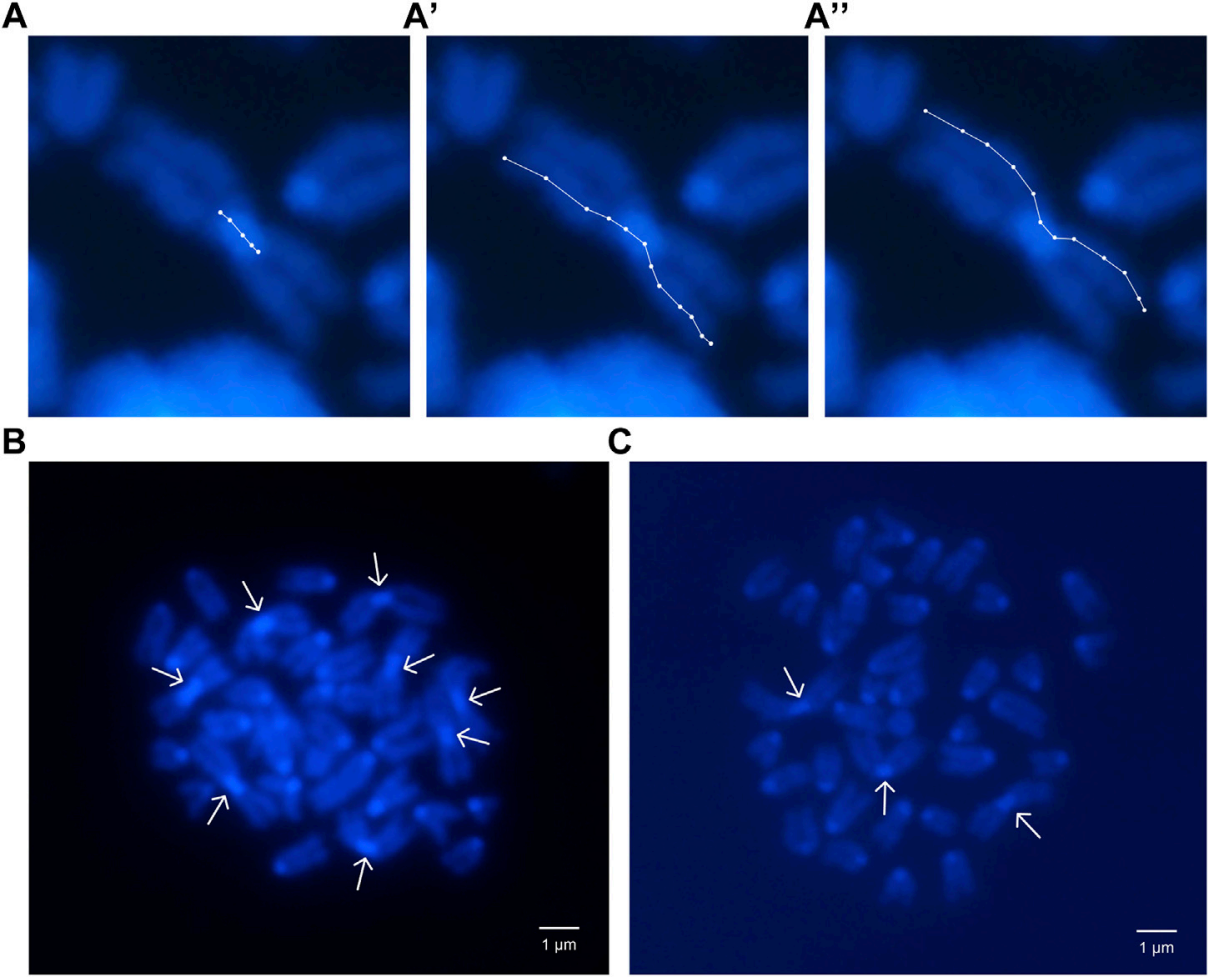
Mitotic chromosomes stained with DAPI. **(A)** Pericentromeric region length of a Rb chromosome; **(A′,A′′)** Sister chromatid lengths of same chromosome. **(B)** Heterozygous parent *2n* = 32 metaphase plate. Arrows show 8 metacentric Rb chromosomes. **(C)** Descendent C, *2n* = 37 metaphase plate. Arrows show 3 metacentric Rb chromosomes.

The size of the pericentromeric regions of the Rb chromosomes was measured in the eight Rb chromosomes of a 2n = 32 heterozygous male, and in the Rb chromosomes inherited by his six sons (Figure 3). The descendants named A, B, C, D, E, and F presented respectively three, five, three, two, four, and five Rb chromosomes and diploid numbers of 37, 35, 37, 38, 36, and 35, respectively (Table 4).

**TABLE 4:** Diploid number, Rb chromosomes and lengths of the pericentromeric regions of Rb chromosomes in the members of a family composed by a multiple Rb heterozygous male and a homozygous female *2n* = 40 and their six offspring. The length of the centromeric region (CL) was measured in pixels and normalized with respect to the chromosomal total length (CL/TLx100).

*Animal*	Diploid number	Number of Rb chromosomes	Pericentromeric length (CL/TL)%
Rb heterozygous male parent	2n = 32	8	21.51%
Descendant A	2n = 37	3	21.11%
Descendant B	2n = 35	5	25.61%
Descendant C	2n = 37	3	21.7%
Descendant D	2n = 38	2	26.28%
Descendant E	2n = 36	4	25.22%
Descendant F	2n = 35	5	22.29%
Homozygous female parent	2n = 40	0	--

The length of the pericentromeric regions was estimated as a percentage of CL to TL (Figure 3; Table 4). In five of the six descendants, the length of the pericentromeric region was greater than the average length of the pericentromeric regions of the Rb chromosomes of the father (Table 5). In the offspring of this family, the ratio 5/6 is greater than 0.5 (inheritance by chance) and *p* = 0.0512.

**TABLE 5:** In 5 of the 6 descendants, the length of the pericentromeric region was verified to be greater than the average length of the pericentromeric regions of the Rb chromosomes of the father. The ratio 5/6 is greater than 0.5 (inheritance by chance) *p* = 0.0512.

Pericentromeric lengths (CL/TL) of descendants%	Father’s Rb pericentromeric region length%	Descendants with greater pericentromeric region lengths
21.11	21.51	0
26.61	21.51	1
21.7	21.51	1
26.28	21.51	1
25.22	21.51	1
22.29	21.51	1

## Discussion

Since the first observation of chromosome behavior during meiosis in the grasshopper *Brachystola* (Sutton 1903; Carothers 1917), the segregation of homologous chromosomes during meiosis is widely considered as being fundamentally random. However, clear examples have been described in various organisms where meiotic drive or distortion with respect to random meiotic segregation occurs (Bongiorni et al., 2004; Presgraves, 2009; Dawe et al., 2018; Malinovskaya et al., 2020; Kruger and Mueller, 2021; Pajpach et al., 2021).

In natural populations of *Mus domesticus*, a totally telocentric ancestral karyotype, Robertsonian translocations or centric mergers occur that originate from Rb metacentric chromosomes and reduce the chromosome number (Pialek et al., 2005). Given the unusual speed with that Rb chromosomes have set in some populations, it has been proposed that Rb chromosomes can be inherited preferentially with respect to the ancestral telocentrics in the descendants of heterozygotes (Britton-Davidian et al., 2000; Britton-Davidian et al., 2005). In these cases, the meiotic drive or preferential segregation of some of the chromosomal forms were observed (de Villena and Sapienza, 2001; White et al., 2010; Clark and Akera, 2021).

Crossroads between both chromosomal forms are found in heterozygotes; in the first meiotic prophase, the metacentric Rb chromosome synapsed with the telocentrics, homologous to their arms, forming a trivalent, and subsequently, must segregate between them (Wallace et al., 2002). Although multiple Rb heterozygotes have a loss of spermatocytes at prophase I and majorly at meiotic divisions produce gametes and are fertile (Garagna et al., 2001b; Wallace et al., 2002; Manterola et al., 2009).

In Rb heterozygous males, gametes produced by alternate meiotic segregation, in which the Rb metacentric chromosome separates from the telocentric chromosomes, seem to predominate (Manieu et al., 2014). In human males heterozygous for one Robertsonian chromosome, 70%–80% of the total gametes are normal or balanced gametes (Wang et al., 2017; Lamotte et al., 2018).

In this study, we analyzed whether Rb chromosomes were preferentially inherited with respect to the telocentric chromosomes, by the offspring of heterozygous males and females for eight Rb chromosomes. Our data showed that offspring inherited between 0 and 8 Rb chromosomes and that the distribution of frequencies was not binomial. The non-binomial distribution of the descendants of any of the heterozygous parents suggests that the number of inherited Rb chromosomes would not be a random process, at least in quantitative terms, because Rb chromosomes have not been distinguished between them. We did not find that descendants of heterozygous females inherited more Rb chromosomes than those of heterozygous males. In contrast, we found that male descendants presented a significantly higher number of Rb chromosomes.

Obviously, these results were obtained from heterozygotes of crosses of the CD1 strain (2n = 40) with the Milano II race (2n = 24), and therefore cannot be directly extrapolated to all the *Mus musculus domesticus* strains/races.

The configuration of the trivalent in the meiotic prophase of Rb heterozygotes could favor the alternating segregation of the anaphase, but this chromosomal configuration could also be relevant in the fixation of the Rb chromosomes in hybrid populations. This could be because this synaptic configuration ensures an obligatory encounter between the Rb chromosome and its telocentric homologous chromosomes, giving rise to the closeness of the abundant pericentromeric heterochromatin of the three gathered chromosomes (Berríos et al., 2014; Berríos 2017). This chromosomal conjunction, which is reiterated in thousands of cells, may also contribute to a second-centric fusion. In addition to the mentioned chromosomal configuration of first meiotic prophase, the intense DNA nicking and repair activity (Neale and Keeney 2006) and the homology of satDNA sequences shared by the mouse telocentric chromosomes create the conditions for new chromosomal rearrangements (Garagna et al., 2001a; Kalitsis et al., 2006). In this way, the concurrence of elements that could favor new chromosome-centric fusions would enhance the chance of those chromosomes regaining the homozygous condition.

The successful fixation of an Rb chromosome in natural populations is the result of the meeting of heterozygotes and the production of homozygous descendants. Any meiotic condition that favors Rb chromosomes in gametes also contributes to the fixation of Rb chromosomes.

The hypothesis that heterozygous females would transfer more Rb chromosomes to their offspring than males arises from findings in different organisms (de Villena and Sapienza, 2001; Fishman and Saunders, 2008; Presgraves, 2009; Dudka and Lampson, 2022). In addition, segregational asymmetry is difficult to explain in males, whereas in female meiosis, such asymmetry is possible because the chromosomes that eventually remain in the oocyte can be inherited, while those that remain in the polocytes are necessarily lost (Lindholm et al., 2016). In this scenario, the differences in centromere strength has been shown to predict the direction of driving. Stronger centromeres, manifested by increased kinetochore protein levels and specific interactions with spindle microtubules, would produce chromosomes that are preferentially retained in the egg (Chmatal et al., 2014; Akera et al., 2017; Wu et al., 2018).

Considering that the main cellular mechanism proposed has been focused on centromere size and its segregational advantage, we found it interesting to show what was observed in the measurement of the pericentromeric and centromeric regions of Rb chromosomes in six descendants of an Rb heterozygote. In mice, the centromeric region contains repetitive centromeric DNA sequences that are enriched in minor satellite repeats, which are prominent sites for centromeric proteins CENP-A and CENP-B assembly (Guenatri et al., 2004; Pajpach et al., 2021). The mouse pericentromeric region contains inactive chromatin and is composed of major satellite repeats, that are required for heterochromatin formation (Guenatri et al., 2004; Pajpach et al., 2021). In 5/6 of the sons, the average size of the centromeric region of the inherited Rb chromosomes was found to be significantly larger than the average size of the same region in the eight Rb chromosomes of the heterozygous male parent. The measurement made involved the centromeric DNA and the abundant pericentromeric heterochromatin located in the proximal p and q regions of the Rb chromosomes. These observations are not comparable with the cellular analyses of segregational efficiency in meiosis of the Rb chromosome according to the size and strength of its centromere because our measurements included the pericentromeric heterochromatin that we do not know how much it may contribute to the meiotic segregation of chromosomes (Chmatal et al., 2014; Iwata-Otsubo et al., 2017). On the other hand, the centromeric region sizes estimated here are on Rb chromosomes that have successfully appeared in the descendants, after overcoming meiotic segregation, gamete differentiation, fertilization, embryonic development and birth. This observation suggests that the larger size of the entire centromeric region is advantageous to the inherited Rb chromosomes.

## Data Availability

The raw data supporting the conclusion of this article will be made available by the authors, without undue reservation.
